# Needs of families of children with intellectual and developmental disabilities in Addis Ababa

**DOI:** 10.4102/ajod.v9i0.735

**Published:** 2020-12-09

**Authors:** Heather M. Aldersey, Ansha N. Ahmed, Haben N. Tesfamichael, Natasha Lotoski

**Affiliations:** 1 Department of Rehabilitation Therapy, Faculty of Health Sciences, Queen’s University, Kingston, Canada; 2 School of Public Health, College of Health Science, Addis Ababa University, Addis Ababa, Ethiopia; 3 Department of Occupational Therapy, Faculty of Health Sciences, Queen’s University, Kingston, Canada

**Keywords:** Addis Ababa Ethiopia, Africa, family needs, family, intellectual and developmental disabilities, spirituality, children

## Abstract

**Background:**

Family support is an essential component of caring for children with intellectual or developmental disability (IDD), however, specific family support needs in developing countries, such as Ethiopia, have received minimal attention in the literature to date.

**Objectives:**

This study sought to understand the specific disability-related support needs of families with children with IDD in Addis Ababa, Ethiopia. We answered the following questions: (1) How do family members of children with IDD in the Mekaneyesus Centre in Addis Ababa currently meet their disability-related support needs?; (2) what are these family members’ most pressing unmet disability-related needs? and (3) how do family members perceive their capacity to meet their support needs?

**Method:**

This study drew from an exploratory qualitative descriptive approach with 16 family members of children with IDD, recruited from a centre for children with IDD. We conducted semi-structured interviews in Amharic. We transcribed and translated interviews into English and guided by a conceptual framework for family support from Kyzar et al. (2012), we thematically analysed the data.

**Results:**

Participants identified instrumental and emotional needs to be most prominent, with additional discussion around various physical and informational needs. Participants identified childcare as the most significant unmet need, which resulted in the loss of various important life roles. The participants discussed major sources of support coming from spirituality, family members and community. Stigma emerged as a critical family support theme external to the Kyzar et al. (2012) classifications of family support.

**Conclusion:**

Although family members are adapting and responding to meet their needs in the best way they can, additional support, particularly related to childcare and future planning, is essential.

## Literature review

Families have the primary responsibility of promoting the overall development and health of their children by fulfilling physiological and emotional needs whilst providing adequate material resources for the child to develop and grow (Berger & Font 2015). For individuals with intellectual and developmental disabilities (IDDs), the family unit is integral to secure opportunities for its members to participate meaningfully (Wingspread Family Support Summit 2011). According to Wang and Brown (2009), previous literature has demonstrated the profound impact of disability on families, and that children with disabilities are served best when professionals collaborate directly with families and provide ongoing family support. We define family support as:

A set of strategies directed to the family unit but that ultimately benefit the individual with IDD. Family support strategies are intended to assist family members, who have a key role in the provision of support and guidance to their family members with IDD. These strategies are designed, implemented and funded in a flexible manner that addresses the emotional, physical and material well-being of the entire family. (Wingspread Family Support Summit 2011:2)

Literature has found that regardless of origin, families of children with disabilities face significant financial and emotional difficulties and are generally dissatisfied with the current disability services and support that they are provided (Wang & Brown 2009). A synthesis conducted by Kyzar et al. (2012) found that family support significantly enhanced family functioning, quality of life, satisfaction and buffering of stress.

Although research has explored the idea of family support in families with disabilities, the data are insufficient as the majority of research was conducted in the United States of America and the United Kingdom (Kyzar et al. 2012; Turnbull et al. 2007; Wang & Brown 2009). Furthermore, the International Association for the Scientific Study of Intellectual and Developmental Disabilities (IASSIDD)Special Interest Research Group on Quality of Life has historically consisted of a diverse cultural group of researchers but without many from countries in Africa (Wang & Brown 2009). Whilst assessments, interventions and necessary services are limited in most of Africa, the majority of research conducted continues to focus on high-income Caucasian families and fails to explore the important aspects of family support in families with disabilities (Malcolm-Smith et al. 2013).

According to Turnbull et al. (2007), the significant lack of diversity in family and disability research is highly problematic and future research must concentrate on the development of collaborative partnerships with participants from culturally and linguistically diverse backgrounds. Although there are often shared experiences that we hear from families across different locations, every country has unique features within its social and cultural contexts that can shape the experiences and support needs of people with disabilities (PwDs) and their family. To provide further insight about common and unique aspects of support, it is critical to understand family-support needs within a given context. For example, we cannot assume that family experiences of disability and support are the same in Addis Ababa as they would be in Cape Town or Accra – as such we need empirical data to better understand and address this issue in a way that is relevant to the local context. The current literature suggests that future family support research must strive for data from non-native English-speaking countries, including fathers and other family members in addition to mothers (Schlebusch, Dada & Samuels 2017) and incorporate specifics of ethnicity, employment and income of participants (Kyzar et al. 2012). Kyzar et al. (2012) also recommend more research about the effects of professional support on families, as the majority of current studies narrow their focus to informal supports, such as friends and family. Despite being few in number, studies in Africa about family-support needs related to IDD indicate challenges of caregiving and a scarcity of support services for both the children and family members (Masulani-Mwale et al. 2016, 2019). These studies portray that having children with IDD often results in marital difficulties, mental health issues and high degrees of stigma from the community. There has been limited research on these issues in Ethiopia.

The United Nations Convention on the Rights of Persons with Disabilities (UNCRPD), which promotes human rights and inclusion of people with disabilities, was ratified in Ethiopia in 2010 (Federal Democratic Republic of Ethiopia 2012), committing the country to uphold the rights of children with IDD for education, safety and inclusion. According to the World Health Organization (WHO) (2011), the prevalence of disability in developing countries such as Ethiopia is 17%, however the exact prevalence of people with IDD in the Ethiopian context is unknown. Individuals with IDD in Ethiopia are supported formally – predominantly through physiotherapists and mental health providers, such as psychiatric nurses and psychiatrists (Tekola et al. 2016). According to Tekola et al. (2016), the expertise and services provided by these professionals are limited to government and private clinics, specialised schools and centres and community-based rehabilitation (CBR) programmes. The focus on these services alone implies a predominantly medical-model approach to IDD in this context, rather than the human rights approach espoused by the UNCRPD.

There is limited existing literature in Ethiopia that explores the impact of support from specialised schools and centres for children with IDD and their families. Tekola et al. (2016) reported that specialised centres are an integral part of service provision in Autism-related services, and this is supported by the experiences of families in Tanzania, as reported by McNally and Mannan (2013). Specialised centres provide a wide array of therapies focusing on social, academic, communicative and activities of daily living (ADL) functioning, in addition to engaging in disability awareness-related initiatives (Tekola et al. 2016). An example of a specialised centre in Addis Ababa, Ethiopia is the Ethiopian Evangelical Church Mekaneyesus Centre for Mentally Challenged Children (EECMY-CMCC), which has provided rehabilitation and education services for individuals with IDD since 1986. An email from the director of the centre (B. Zurgie, pers. commun., 10 March 2020) confirmed that the centre provides services for individuals from 3 months old to adults, and the services provided include early intervention, Montessori classes, pre-vocational and vocational classes for about 400 children with various disabilities (i.e. mainly children with IDD, cerebral palsy, spina bifida and malnutrition).^1^ The Centre provides support at no direct cost to the family. Beneficiaries of the centre are from diverse religious backgrounds and the function of the centre is independent from the church-based services; however, the management system and financial support are under the church.

This study aims to describe how family members of children with IDD in the Mekaneyesus Centre currently meet their disability-related support needs. By exploring the met and unmet needs of family members, we gain a deeper understanding of the lives of the families and caregivers in an Ethiopian context. Specifically, we sought to answer the following questions: (1) How do family members of children with IDD in the Mekaneyesus Centre in Addis Ababa, Ethiopia currently meet their disability-related support needs?; (2) what are these family members’ most pressing unmet disability-related support needs? and (3) how do family members perceive their capacity to meet their disability-related support needs? The results provide insight into the experience of family members of children with IDD, and help to create an understanding about their needs in the context of a developing world.

## Conceptual framework

Kyzar et al.’s (2012) synthesis of the global family support literature organises results based on four types of support: emotional, physical, instrumental and informational. Emotional support refers to any support that results in better affective and psychological well-being by reducing stress and other negative emotions. Physical support entails assistance in day-to-day functioning, including ADLs. Instrumental support refers to assistance in terms of access to financial resources and undertaking important duties of the family, such as childcare. Informational support refers to the provision of information in different formats to make informed decisions about the disability (Kyzar et al. 2012). This study used the Kyzar et al. (2012) classifications of support to assist in framing the interview questions. We also used this to help shape preliminary analytical discussions around the findings. Although we viewed our findings in light of this pre-existing classification, we were also attuned to any results that emerged inductively that were relevant to the research questions but not wholly in alignment with the existing framework.

## Methods

We conducted this study using an exploratory qualitative descriptive approach (Sandelowski 2000). Specifically, we recruited 16 family members from the Mekaneyesus Centre for Mentally Challenged Children in Addis Ababa, Ethiopia to participate in semi-structured interviews. Participants were included if they were: (1) 18 years of age or older, (2) self-identified as a family member of a child with IDD and (3) were able to converse in Amharic. We excluded participants who were caregivers of children with IDD but who did not self-identify as a ‘family member’. We recruited participants purposively, striving for diversity in representation of gender (both male and female), socioeconomic status (low, medium, high), education status of participant, family role (e.g. not just mothers) and nature or severity of the child’s disability.

Firstly, all potential participants who met the study inclusion criteria were identified through discussion with the centre director, teachers and physiotherapy professionals working in the centre. Once potential participants who might meet the recruitment criteria were identified, the centre director approached them to inquire about their willingness to speak with the data collector for research related to their child or family member with a disability. Parents who indicated an interest to participate and who met the desired individual characteristics of our purposeful sampling were contacted by a researcher to explain the study and the content of the informed consent form. All participants were given the opportunity to choose where they would like the interview to take place, with all electing for a quiet and separate room at the Mekaneyesus Centre. In total, we approached 17 interested individuals and one individual was ultimately unable to join the study because of scheduling conflicts during the time of data collection. All participants provided written informed consent prior to the commencement of the interview. Participants did not receive compensation for their participation in the study. We conducted all interviews in Amharic using a semi-structured interview guide. We also asked preliminary demographic information of the families prior to commencement of the study. It is important to note that although Centre staff identified people who had children with IDD as potential participants, we asked the family members themselves to characterise what they believed to be the severity of their child’s disability once they agreed to take part in the study. We did not evaluate or assess official diagnoses. Each interview session was audio-recorded and ranged from 30 to 64 min. Table 1 provides the semi-structured interview guide used in the interviews.

**TABLE 1:** Interview questions.

Number	Question
1. Could you please tell me about your family? Probe: Who lives at home? Tell me about your child with a disability?
2. What does your family enjoy doing together?
3. Often, families who experience disability need support to best care for their member with a disability. Could you please tell me about any disability-related support needs that your family experiences? Probe: Emotional, instrumental, material and physical Probe: Are these needs currently met or are they unmet?
4. What do you do (or where do you go) to meet your family’s needs/get the support that you need?
5. What support do you wish you had available to better meet your family’s needs, but that isn’t available to you right now? Probe: Who should provide these?
6. Describe the current capacity of your family to meet the disability-related needs to support your child.
7. Is there anything else that you would like to share with us related to your family’s support needs and priorities for the future?

Once the primary data collector, with another co-author, believed that the study had reached adequate information saturation, she then interviewed two more participants for better confidence and stopped after the 16th participant. We transcribed interviews verbatim into Amharic and Amharic transcripts were later translated into English by two bilingual experts, following appropriate procedures for data protection and confidentiality. We imported all transcripts into NVivo12 qualitative data analysis software and coded thematically using Braun and Clarke’s (2006) approach to thematic analysis. Two authors coded the Amharic transcripts directly and two authors coded the English translations of the transcripts. We coded both deductively based on the Kyzar et al. (2012) classifications and inductively to identify any themes that might fall outside the classifications. All authors met regularly to discuss and agree on a coding guide and, ultimately, to harmonise and finalise emerging themes.

### Ethical consideration

Ethical approval to conduct the study was obtained from the General Research Ethics Board, Queen’s University, reference number: GRHBS-123-19.

## Findings

A total of 16 participants took part in the study, including nine mothers, five fathers, one adoptive mother and one aunt of a child with IDD. Table 2 gives additional information about the demographics of the participants. Given our limited capacity for diagnosis or assessment, we included participants who self-identified as having a family member with IDD and used self-reports for the severity of the disability (i.e. we did not assess the disability of the family member in this study).

**TABLE 2:** Participant demographics.

ID	Nature or severity of disability (self-report)	Age of child with a disability (CWD)	Relationship with CWD	Marital Status	Family members	Monthly income in Birr (and USD equivalent)	Highest level of education attained
P1	Severe	6	Father	Married	4	Missing	Grade 1
P2	Mild	6	Father	Married	4	~4000 (116.59)	Diploma
P3	Severe & Mild (2 children)	6 and 2	Mother	Married	7	~4000 (116.59)	No school
P4	Mild	29	Mother	Married	6	48 510 (1413.99)	1st degree
P5	Mild	7	Mother	Married	6	~2000 (58.30)	Grade 10
P6	Mild	6	Mother	Married	3	Not consistent	Grade 10
P7	Mild	12	Mother	Single	4	Not consistent	Missing
P8	Mild	18	Adoptive Mother	Married	3	~3600 (104.93)	Grade 12
P9	Mild	7	Aunt	Married	10	Not consistent	Grade 6
P10	Mild	11	Mother	Single	2	Not consistent	Grade 5
P11	Mild	15	Father	Married	7	~11 000 (320.63)	1st degree
P12	Mild	20	Father	Married	6	~15 000 (437.23)	Grade 9
P13	Mild	5	Mother	Single	2	~1200 (34.98)	Grade 8
P14	Severe	9	Mother	Single	3	Missing	Grade 8
P15	Mild	6	Mother	Single	6	~1720 (50.14)	Grade 12
P16	Severe	4	Father	Married	4	~2500 (72.87)	Grade 7

Note: The Ethiopian birr (ETB) was converted to USD by the currency rate around the time of data collection at which 1 ETB was 0.029 USD. According to World Bank report, the per capita income of Ethiopia in 2018 was 772.3.

### Family support needs

As stated above, the categorisation developed by Kyzar et al. (2012) guided the conceptual framing of the identified family support needs of participants in our study. Whilst this framework provided a structure for the organisation of our themes, it is important to acknowledge that whilst we tried to distinguish between them, the identified needs were, sometimes, significantly intertwined – for example, needs were interrelated within categories (e.g. not having childcare affected the family’s finances) and across categories (e.g. families needed emotional supports because of burden created by instrumental-support needs). We have also identified one theme (stigma) that might not be fully captured in the Kyzar et al. (2012) classifications.

#### Instrumental

Instrumental needs such as childcare and workplace flexibility, financial support, transportation and future planning were the most prominent themes in the data.

#### Childcare

Every participant stated in some form or another that the childcare available in Addis Ababa was insufficient for children with disabilities. Participants explained that there is simply a lack of childcare centres for children with disabilities, and that the existing centres are either over their capacity, or geographically and financially inaccessible.

‘The important thing is there are no day-care centres. There are not even private day-care centres that we could pay for. No matter how much money you are willing to pay, there are no centres that take [*a child with*] such a case and give treatment also.’ (Participant 2)

Because of the significant lack of childcare in Addis Ababa, participants stated that they are often unable to fulfill their responsibilities as workers and students, missing school and work to care for their children. Many participants explained that they had to quit their jobs in order to meet the care demands of their child.

‘I had planned to study nursing but suddenly [*my child*] became sick and she was admitted to Yekatit [*hospital*] for 10 days. So I missed the registration for the school and just attended to the needs of my child. When I went there, they told me that the registration deadline had passed … we all started to think about what will happen if she becomes sick when I go to school; so we all agreed that I need to stay at home and take care of her until she reaches school age.’ (Participant 15)

‘My wife had a profession but she quit work because of our child. She [*my wife*] is now a housewife … She studied computer science and she used to work at an NGO. After she [*our child*] was born, our job became to wander around looking for services.’ (Participant 11)

Some participants asserted that managing their responsibilities as an employee was extremely difficult whilst having a child with a disability. Many participants expressed that their workplaces were not flexible in supporting their unique needs and they mentioned their need to get a job that can enable them to work whilst still providing care for their child with disability (e.g. workplace daycare or work done alongside the child). In fact, many participants stated that their employers terminated them when they requested time off to attend necessary medical appointments for their children. For example, Participant 1 was terminated after being 10 min late for work whilst taking the child to the hospital.

Some participants suggested that the government has the responsibility to meet the childcare and unique service needs of families with disabled children in order to facilitate their ability to maintain employment. For example, 10 of the 16 participants were adamant that the government could provide better childcare services for children with IDD.

In addition to the impact on work and school roles, lack of childcare also significantly affected the social roles of some participants. These participants explained that they were required to reduce their participation in social activities in order to stay home and care for their child.

‘… After stopping working … [*my wife*] is living with psychological stress. Even if you have nothing to eat or drink, just spending some time with others helps you to withstand all this. It has something good. But after this child was born, it is only this centre that she comes to. She has no other place to go. Her friends who used to call her have disappeared and her families are in the countryside.’ (Participant 16)

The participants explained that whilst many of them gave up important life roles to stay home and care for their child, there are still times in which they must leave the house, in which they often make the difficult choice of leaving their child alone at home. This results in fear and concern for participants, in regard to the safety of their child.

‘Sometimes when I come here, I have to restrain my child and leave him alone. You could not ask your neighbours to look after him. If my son was well, you could leave him with neighbours … But when I come here, I am so worried whether he falls out of his wheelchair, or gets electrocuted.’ (Participant 14)

Whilst many participants expressed that their unmet-childcare needs result in personal sacrifice of productive roles in the community, it must be stated that some participants expressed that the centre met their unique childcare needs. Additionally, participants stated that their family members as well as community members often provided childcare support.

#### Financial support

Ten participants explained that they experienced significant financial hardship because of unmet financial-support needs. These participants explained that having a child with IDD demands additional expenses for necessary medication and treatment. Participants explained that they spend the majority of their income on their child with IDD.

‘We spent all the money that we had at hand for the medication, in the end we remained empty handed.’ (Participant 1)

Whilst the majority of participants expressed financial difficulties, they stated that when these financial needs were met, they were often met by their extended family members. Other participants mentioned explicit acts of kindness by community members such as taxi drivers, hospital workers and fellow church-goers, who informally gave them money to help with costs related to the child with IDD.

‘It is my sister. I live on her … We have nothing other than my sister’s support. She shares with us from her limited income and resources.’ (Participant 14)

‘It is the passersby that bought him milk when they saw me walking carrying him. Everyone would like to help me pay for our taxi expenses when we travel.’ (Participant 1)

#### Transportation

The theme of transportation arose from the data as an important unmet instrumental need, referring to the difficulties that participants were having in transporting their children to the centre. Nine participants explained finding transportation to specialised schools or centres in Addis Ababa was extremely difficult and inconvenient for their daily schedule, as it was unaffordable and both geographically and physically inaccessible. One participant explicitly stated that she could not use public transportation because it was not wheelchair accessible. Participants reported that when they could not meet their transportation needs, they carried their children significant distances by foot in order to ensure attendance at the centre.

#### Future planning

Ten participants identified planning for the future as another important unmet instrumental need. Planning for the future refers to arranging support and plans for the children as they surpassed the age of 18 and as the participants themselves became less able to care for them. Participants indicated that existing services are available for children, but the service options became quite sparse when children surpassed the age of 18.

‘Until 18 or 20 years old and up to that they will have training. After that they will leave the centre but what will be done after he leaves the centre … What will I do after he becomes 18 years old? I have no capacity to do anything. Usually, I am worried about it.’ (Participant 5)

The participants explained that there is a complete lack of financial security for the future of their children. They explained that this resulted in feelings of worry and fear in regard to the future trajectory of their children.

Whilst participants explained that planning for the future was an unmet support need, they did express interest in forming regular meetings with other families experiencing the same situation. Participant 14 stated that families with children with similar disabilities formed a peer support organisation that hoped to register legally and create small businesses to support themselves.

#### Emotional

Many participants demonstrated unmet emotional needs, as indicated by feelings of hopelessness. They expressed a profound amount of stress and difficulty resulting from having a child with a disability, and this resulted in unique emotional needs. These participants expressed a loss of hope, faith and aspiration following the birth of their children with IDD. One participant explicitly stated that he had attempted suicide because of the situation for which he sought treatment and counselling.

‘Your mind becomes sick, as a human being you lose your hope….’ (Participant 6)

‘After this child came to our life, things are not easy. I am overwhelmed.’ (Participant 9)

Following the birth of their disabled child, mothers, in particular, indicated that their emotional needs were not met. Four mothers shared that their husbands left them because of the emotional struggles faced from having a child with a disability.

‘I suffered a lot. Even my husband assumed that as if I created her with my hands [*am the reason for the disability*]. I had a lot of challenges. Finally he walked away.’ (Participant 6)

‘Yes, my husband isn’t there … He abandoned me because of the child … On the 14th day after the child was born, he was asked to donate blood to the baby, he refused saying why do I give blood for a child that couldn’t recover … After that, what kind of life can you have with this person? I lost all of my hope in him.’ (Participant 14)

Furthermore, many participants indicated that their extended family members did not meet or support their emotional needs. These participants stated that they were ridiculed, discriminated against or witnessed blatant insults directed at their child.

‘My family told me not to bring her [*my child*] to their house … You may not believe this but when I took her there one day, my brother saw her and said, “you idiot – you came here” … I will never forget that time. I just went to another room not to create more mess.’ (Participant 6)

Most participants consider peer support groups to be important in helping the family members cope through difficult times. They mentioned the importance of discussion and sharing of their ideas and experiences with other family members. However, our results demonstrated that the family members used peer-groups to meet their instrumental support needs more than their emotional needs.

Conversely, some participants, however, explained that their immediate and extended families did sufficiently support their emotional needs.

‘I tell her [*my wife*] that the money that we get by both of us working might not be blessed [*although being plenty*]. On the other hand, even with only my salary, we are missing nothing. We are leading our family and we never get hungry or thirsty. We need to understand that. We don’t know if a person that is affluent has what is in us. We need to be thankful as we are healthy and living with what we have.’ (Participant 11)

Other participants explained that connecting with others in the community and sharing their stories helped them to meet their emotional needs. Healthcare professionals also provided some participants with feelings of hope. Despite the stigma surrounding them, many participants in this study focused on asserting themselves in their narratives, indicating that this made them feel better about their situation.

‘There are mothers that remain at home. I have already come out, so I have nothing to be afraid of or be ashamed of. I would be happy if the majority of mothers come out to the community and are seen by the public. We shouldn’t get ashamed of it because it is God’s will and whether we are ashamed or not, nothing changes. It never changes. Before I used to feel ashamed, but I don’t care about that now.’ (Participant 10)

The preceding quote also demonstrates the remarkable role that spirituality has in meeting the emotional needs of participants. All 16 participants stated that spirituality helped them to cope with their situation of having a child with IDD. These participants explained that their spirituality and religious beliefs in a higher power helped them maintain a positive outlook on their situations. Additionally, several participants believed their child was brought to them by God, and that therefore they must be thankful.

‘I tell my family or the community that this is what God gave me. It is not me who brought it. If God wants, he can give it to everybody by knocking their door. This is how God tests his own people. If I can’t stay strong, I will not be blessed. I don’t bring it in purpose but it’s God who gave me. You must not be a person who is thankful when getting his wishes and complaining when things do not go his way … It is God who he knows what may happen tomorrow.’ (Participant 14)

‘We laugh at everything. We laugh loudly. People say what makes you laugh like this since you have two disabled children. I replied to them that my fate is in the hands of God. You can’t inhibit me from laughing because God himself will stretch his hands to my children.’ (Participant 3)

#### Informational

Many participants stated that they were unaware of many, if any disability-related services for families with children with disabilities, indicating an unmet informational support need. Eight participants explicitly stated that they heard of the Mekaneyesus Centre by chance, by word of mouth. Participant 2 was the only participant to state that the government had recently created a disability awareness campaign in which wheelchairs were also provided to very few individuals with mobility issues. One participant mentioned that a community-based rehabilitation worker provided the information about an available service.

‘Some people tell me there are organisations everywhere that give support. They asked me whether I received support from three organisations … God is my witness that I know no such places. Even I brought him here when a health extension worker who was providing house to house vaccination saw him and insisted that I should bring my child here.’ (Participant 1)

In terms of information related to diagnosis of disability, some participants stated that this information was promptly and accurately provided by healthcare professionals, whilst others explained that receiving the information took a long time and was inaccurate. Two participants (Participants 6 and 15) explained that it could take up to 4 years to receive a diagnosis. Conversely, three participants (Participants 9, 14 and 16) explained that they were given diagnostic information immediately upon being in contact with a health professional.

#### Physical

Physical needs of participants are tangible items such as medications, treatment, food and support with ADLs. Many participants mentioned a plethora of unmet physical needs.

Whilst participants identified an extensive range of unmet physical needs, some stated that their needs were met by support from their families as well as the centre itself. Participants explained that their families often provide help with ADLs and the centre provides food and other material goods. Furthermore, the government provided some essential equipment in the treatment process that fulfilled the needs of family members.

‘My family may God bless them. My sister especially never distastes him. When she comes to my home, she washes and dresses him. She makes him look good. No disregarding at all.’ (Participant 14)

‘I cannot say government doesn’t support us at all … the wheelchairs were given to woreda^2^ and for very few people with severe mobility problems.’ (Participant 2)

#### Stigma – External to Kyzar et al. (2012) framework

As previously stated, we organised our data through the needs framework outlined by Kyzar et al. (2012); however, our data shed light upon a theme, stigma, which could not easily be included in one of the four categories of family needs. It is important to highlight the theme of stigma as an individual theme because it had a profound impact on the lives of many of the participants we interviewed, and helps us to understand family needs in this context.

Twelve participants expressed facing significant stigma in their lives, manifested in different forms. They expressed a need to reduce the stigma that they experienced as a result of the family member with IDD. Some participants felt stigma in the form of exclusion from community events, whilst others explicitly stated that neighbours told their children to stay away from and not play with children with disabilities. For example, one participant (Participant 14) described an incident in which her son was excluded from a birthday party because of his disability.

‘My social life after I gave birth to him [*son with IDD*] reduced a lot. My neighbor had a birthday party and she said send your daughter after dressing her … She said send your daughter not send your children.’ (Participant 14)

Other participants explained that the stigmatisation and discrimination they faced were a result of commonly held beliefs within the community in regard to disability, including the idea that disability is a curse or is created in response to a sin. Stigma affected family needs in a range of ways – including related to creating distress for the family and need for emotional support, and affecting a family’s ability to use public transportation or meet their financial needs.

## Discussion

Our study illustrates the plethora of family support needs of families with children with IDD in Addis Ababa, Ethiopia. Through the application of the Kyzar et al. (2012) support classifications, we were able to organise the various needs as emotional, informational, instrumental and physical support needs, and we added the stigma classification. The most prominent themes that arose from the data were related to instrumental and emotional support needs. The instrumental family support need of childcare was unquestionably the most significant unmet need, resulting in caregivers sacrificing their own employment or education. In addition to childcare, transportation and future planning were significant unmet instrumental family support needs. The lack of sufficient means of transportation within Addis Ababa prevented many families from receiving necessary disability-related services, whilst the inadequate provision of future planning services for families and children with IDD produced feelings of uncertainty and fear related to the unknown life trajectory of their child. In regard to unmet emotional needs, participants described that having a child with IDD combined with limited instrumental support left them with extreme levels of hopelessness and stress. However, many participants explained that their family members and strong spirituality helped them to manage these significant emotional support needs. It is clear from this description of needs that the categories are highly interrelated (e.g. limited instrumental support resulted in further emotional support needs).

The findings of this study provide further evidence that although there are unique aspects of the family experience of disability in Addis Ababa, many of the family support needs and experiences are highly similar to those of families all around the world. This provides further substantiation that the Kyzar et al. (2012)’s classifications for support are globally relevant for families despite the support context. Furthermore, we believe that stigma is a universal challenge for families of children with disabilities, although it may be enacted and experienced in different ways across cultures. From a theoretical perspective, our results align with various global understandings of disability. For example, as it relates to the International Classification of Functioning (ICF) (WHO 2001), our findings demonstrated that family members experienced a range of restrictions to their participation in daily activities. Moreover, from a social-model perspective (Oliver 2013), families were effectively disabled by a society that was not accommodating their specific needs as it related to transportation, childcare and workplace accommodations.

The significant lack of childcare services and facilities within Addis Ababa was the most pressing unmet disability-related support need of families with children with IDD. In terms of childcare, it is imperative to consider approaches to reduce the caregiving responsibilities of family members. The government could contribute either by developing childcare services in close proximity to family homes, or by advocating for the inclusion of childcare services within workplaces. As many of our participants agreed, they are willing to assist in sharing the responsibility of childcare with whatever organisation takes the responsibility of establishing a childcare service. This sort of peer-support arrangement, where families work together to support one another to provide childcare is a potential support intervention to explore. It is also important to reflect on the fact that many participants advocated for special childcare, specific to children with disabilities. This may be because we recruited participants who were currently receiving support at a segregated centre. It is, however, important to note that providing separate day-care centres for children with disabilities runs counter to global calls for inclusion of children in mainstream settings, and may actually contribute to increased stigma for these children and families. In exploring and advocating for solutions, we would encourage families, support providers and the Ethiopian government to explore how children with disabilities could be better accommodated within existing mainstream childcare options.

The effective use of existing resources facilitated through community-rehabilitation workers working in rural areas could help alleviate the childcare issues faced by families. Community-based rehabilitation is defined by the WHO, International Labour Organization (ILO) and United Nations Educational Scientific and Cultural Organization (UNESCO) as a community development strategy in which rehabilitation, provision of equal opportunities as well as social inclusion are the central goals for individuals with disabilities (WHO, ILO & UNESCO 2004). Community-based rehabilitation is implemented through the collaboration of individuals with disabilities and their families as well as community organisations, the government, educational, vocational and social services (WHO et al. 2004). Additionally, CBR may provide peer support groups, which were of interest to this study’s participants, and which may be one way to begin to address the issue of childcare support.

Beyond childcare support, families expressed that sharing their personal experiences with other families experiencing the same situations would help them learn from one another, but they explained that there is a significant lack of existing peer support groups. Literature on the continent of Africa (and globally) has identified a number of strategies for how families can be better supported. These include parent- caregiver- or family- advocacy and support groups, family income generation and poverty alleviation and community mobilisation (Aldersey, Turnbull, & Turnbull 2016; Bunning et al. 2020; McConkey, Kahonde & McKenzie 2016; McKenzie & Chataika 2018). Studies, such as that by Marimbe et al. (2016) in Zimbabwe examined needs for family caregivers of people with mental health issues and found that peer support groups provided families with opportunities to support one another and enhance coping abilities (Marimbe et al. 2016). Peer support groups allow for interaction and sharing amongst family members confronted with similar caregiving challenges (Mittelman et al. 2006). We believe that many of these approaches would also be relevant ways to increase support for the families depicted in this study.

Community-based rehabilitation (CBR) programs may also be a way to improve support for families in this context as well. A qualitative study by Hansen, Musonde and Van der Veen (2014) examined the perceived support that mothers of children with disabilities in Zambia received from CBR programmes. They found that the mothers in their study appreciated the CBR services they received because of the support provided in the areas of social participation, mobility, provision of equipment and educational and emotional support (Hansen et al. 2014). Mothers explained that the CBR programmes provided ongoing emotional and financial support groups which were extremely beneficial, and that CBR was looked to as having an advocacy role in terms of advocating for accessible schools and financial support in order to fund the education of their children (Hansen et al. 2014). Other studies from various parts of Africa, such as Hartley et al. (2005) in Uganda, have found that caregivers identify educational opportunities for their children with disabilities as a significant need, however the financial and geographical inaccessibility of the available schools results in these needs being unmet.

As it relates to currently meeting their needs, it is notable that spirituality emerged as the most prominent way in which families presently meet their disability-related support needs. Participants discussed spirituality in a wholly positive light in this study. We believe that the significant degree of reliance on spirituality highlights the coping abilities of these families. As described by Krupa et al. (2016), coping is a self-regulating process of managing adversity and demands that exceed an individual’s abilities. When individuals cope, they overcome psychosocial disturbances in order to navigate and implement their own resources to adapt to challenges (Abiola, Udofia & Abiola 2011). Across the literature, the use of spirituality as a positive coping mechanism has been well documented amongst families and caregivers of those with disabilities (Hatun et al. 2016; Masuku & Khoza-Shangase 2018; McNally & Mannan 2013). Beighton and Wills (2017) found that the use of spirituality as a coping mechanism provided parents with feelings of comfort because of having faith that God had purposely gifted them a child with a disability. Furthermore, families often believe that because their disabled child is a gift from God, it is their moral duty to care for them (Hatun et al. 2016). In an African-specific context, spirituality has been expressed as a strong belief in a higher power which allows families of children with disabilities to interpret and cope with their situation in the way of understanding it as out of their immediate control, but rather in the hands of God (Masuku & Khoza-Shangase 2018; McNally & Mannan 2013). Studies specific to Ethiopia, such as that by Fenta and Boon (2018), have stated that many individuals believe that their lives are under the control of a higher power. We believe that the positive coping strategy of spirituality amongst our participants is one aspect of their resilience, which is explained to be the state of optimal self-regulation through successful and effective coping (Compas et al. 2001). Because of the significant attention our participants brought to the importance of spirituality, we believe that future research should explicitly explore the role of spirituality in relation to coping with unmet family support needs of families with children with IDD in developing countries.

The families in this study demonstrated various strategies that they used to support their family needs. They discussed coping with the immense psychological stress resulting from caring for children with IDD and managing to navigate the external factors in order to acquire money, treatment and resources for their children. In a community with scarce availability of childcare, transportation and treatment services, the families persevered to care for their children utilising their own resources as well as whatever help they could obtain from their families, communities and health professionals. The participants sacrificed their own important roles and managed to live in spite of the stigma that they faced, reiterating the findings of Hartley et al. (2005), and most of them demonstrated the willingness to become active agents in their lives to provide for themselves. Our study also aligns with the work of Green (2007) who demonstrated that caregivers with children with disability sacrifice their own time and needs in order to meet the needs of their child, restricting their own participation in social, leisure and employment opportunities which do not accommodate their diverse needs. Whilst they identified a profound amount of unmet financial support needs, most of the participants did not communicate a desire for charity or pity from others. Rather, they demonstrated the desire to work and gain their own financial capital, as portrayed by efforts to establish peer organisations. The majority of participants stated strong aspirations for paid employment, but explained that the insufficient provision of childcare services in Addis Ababa restricted them. Other studies have portrayed the impact of caregiving in restricting family members from engaging in livelihood and social activities (Dogbe et al. 2019; Paget et al. 2016).

The scarcity of childcare for children with disabilities in Addis Ababa seems to exclude a number of individuals from financially contributing to the Ethiopian economy. Respite care has been identified as one possible solution to alleviate the burden of care on families with family members who have disabilities (Van Exel et al. 2006). The introduction of respite care within Ethiopia could, therefore, be a potential way to support families with children with IDD. Future studies should explore childcare solutions for families in this context. In particular, it would be interesting to understand to what extent mainstream pre-schools, nurseries and schools accept these children into their classes and if staff have the appropriate skills and attitudes to do this. If there are gaps in inclusion practice, future advocacy may seek to find ways for these children to be included and appropriately supported in these settings.

Although we attempted to organise our data according to the Kyzar et al. (2012) classification of needs, it is critical to acknowledge that the theme of stigma emerged outside (and seemed to cut across) the four main categories of support needs. Many participants stated that the misconceptions surrounding the origin of IDD resulted in discrimination from participating in productive and societal roles. Stigma in our study was a cross-cutting issue and was reflected in negative societal responses as families attempted to navigate their support needs such as taking public transportation or trying to access childcare. The findings from our study regarding stigma are not unexpected, as existing disability literature has demonstrated a relationship between disability and stigma in high- middle- and low-income countries. In the African context specifically, McNally and Mannan (2013) found stigma and discrimination demonstrated against children with IDD in the form of laughing, staring or avoiding the child altogether. Aldersey (2012) uncovered narratives of support for killing a child with IDD because of community stigma in Tanzania. Within Ethiopia itself, family members of children with disabilities have reported withholding their child’s condition from society because of the stigma they faced, resulting in heightened feelings of stress and depression (Tilahun et al. 2016). Stigma is a critical issue that complicates discussions of family support, and future research should explore the relationships between stigma and family support more deeply.

Finally, we strongly believe that it is not simply enough to identify the needs of families, rather we see this study as the first step in a longer-term engagement with the Centre and the families it supports. Our initial plans to act on study findings had to be postponed because of the COVID-19 pandemic; however, we are actively working to initiate further participatory action projects with direct engagement of participants as soon as it is safe and appropriate to do so.

## Limitations

This study was conducted in one centre in Addis Ababa, and therefore the findings may represent the family support needs explicit only to this area and may not be generalisable to the whole population of Ethiopia. For example, family members in rural and semi-urban areas of Ethiopia may, because of their smaller locations that might allow a greater development of community, experience less need for support around access to childcare and transportation. Future studies should, therefore, explore the family support needs of children with IDD in diverse rural and urban settings within Ethiopia. Moreover, this study included families who were already accessing some support at a centre. Future studies might also include the perspective of families outside formal support settings for greater insight.

Additionally, this study relied on external characterisations of the severity of the family member’s disability; therefore, this classification is subjective. However, the purpose of this study was not to assess or diagnose children with IDD, and we believe that the subjective classification of the severity still gives useful insight into the family situation, as the family perceives it. Study participants had family members with a great variability in age – future studies with more tightly defined inclusion criteria for ‘family member’ may have resulted in more nuanced findings specific to a particular age group.

Additionally, we relied on the Centre staff to identify potential family member participants. Therefore, there is a risk that there may have been some bias that we were not able to identify at the level of family identification and recruitment for the study, however, we do not believe this to be the case, based on the varied participants provided who had varying degrees of communication ability and levels of criticality.

Finally, it is important to reflect on the potential limitation that language may have for this study. Although the interviews were translated and transcribed by two Amharic-speaking individuals, there is always potential for a loss of meaning in translation. Therefore, it is possible that some ideas may not perfectly translate to English. Nevertheless, we have intentionally embedded language considerations throughout this study (e.g. having the two Amharic speakers code in the source language and using this as a further check on the English translations) to reduce the potential limitation that might arise from challenges in translation.

## Conclusion

This study has demonstrated that the instrumental need of childcare is the most pressing unmet need amongst family members at the Mekanyesus Centre, followed by transportation and future planning. Unmet emotional support needs resulted in stress and hopelessness for some participants but many participants met their needs through strong spirituality and support from family. The remarkable influence that spirituality had on all 16 participants cannot be overstated, and this finding correlates with previous literature regarding resiliency. Although facing a wide array of hardships, including significant stigma within their community, study participants demonstrated a strong desire to meet their family needs and contribute to society. As such, many families framed support as a tool that would further enable them to actively meet their own responsibilities of contributing to their family and society (rather than support as a handout). Based on participants’ perspectives, priority family support may take the shape of childcare centres that provide future planning information as well as peer support groups for families with children with IDD in Addis Ababa, Ethiopia. The findings of this study also support the development of policies to improve transportation and workplace flexibility, and introduce respite care to better meet the needs of families of children with IDD. In accordance with the Ethiopian government’s commitment to the UNCRPD, these family perspectives demonstrate that there are important actions that the government and community could take to better promote and uphold the rights of children with disabilities.

## Footnotes

1 In this study, families used various terms to describe their child with IDD, for example, just impairment_ ‘Gudat’ or in a way that is related to IDD, a mental health problem/psychiatric condition/illness ‘Aemiro zigmet or Himem’ and sometimes they would speak specifically of Autism and ‘Aemiro zigmet’ when referring to their child’s disability.

2 Woreda: the name of a geographic location in Ethiopia that is part of a region, but has smaller towns within it.
